# Plasma biomarkers of endothelial function, inflammation and oxidative stress in individuals with non‐freezing cold injury

**DOI:** 10.1113/EP090722

**Published:** 2023-02-20

**Authors:** Clare M. Eglin, Jennifer Wright, Anthony I. Shepherd, Heather Massey, Sarah Hollis, Jonathan Towse, John S. Young, Matthew J. Maley, Stephen J. Bailey, Chris Wilkinson, Hugh Montgomery, Michael J. Tipton

**Affiliations:** ^1^ Extreme Environments Laboratory School of Sport Health and Exercise Science University of Portsmouth Portsmouth UK; ^2^ Regional Occupational Health Team (ROHT) Catterick Catterick Garrison UK; ^3^ School of Pharmacy and Biomedical Sciences University of Portsmouth Portsmouth UK; ^4^ National Horizons Centre Teesside University Middlesbrough UK; ^5^ Environmental Ergonomics Research Centre Loughborough School of Design and Creative Arts Loughborough University Loughborough UK; ^6^ National Centre for Sport and Exercise Medicine School of Sport Exercise and Health Sciences Loughborough University Loughborough UK; ^7^ Department of Medicine University College London London UK

**Keywords:** cold injury, endothelial function, endothelin, interleukin, nitric oxide, oxidative stress, pathophysiology, syndecan

## Abstract

Plasma biomarkers of inflammation, oxidative stress, endothelial function and damage were examined in 16 individuals with chronic NFCI (NFCI) and matched control participants with (COLD, *n* = 17) or without (CON, *n* = 14) previous cold exposure. Venous blood samples were collected at baseline to assess plasma biomarkers of endothelial function (nitrate, nitrite and endothelin‐1), inflammation [interleukin‐6 (IL‐6), interleukin‐10 (IL‐10), tumour necrosis factor alpha and E‐selectin], oxidative stress [protein carbonyl, 4‐hydroxy‐2‐nonenal (4‐HNE), superoxide dismutase and nitrotyrosine) and endothelial damage [von Willebrand factor, syndecan‐1 and tissue type plasminogen activator (TTPA)]. Immediately after whole‐body heating and separately, foot cooling, blood samples were taken for measurement of plasma [nitrate], [nitrite], [endothelin‐1], [IL‐6], [4‐HNE] and [TTPA]. At baseline, [IL‐10] and [syndecan‐1] were increased in NFCI (*P* < 0.001 and *P* = 0.015, respectively) and COLD (*P* = 0.033 and *P* = 0.030, respectively) compared with CON participants. The [4‐HNE] was elevated in CON compared with both NFCI (*P* = 0.002) and COLD (*P* < 0.001). [Endothelin‐1] was elevated in NFCI compared with COLD (*P* < 0.001) post‐heating. The [4‐HNE] was lower in NFCI compared with CON post‐heating (*P* = 0.032) and lower than both COLD (*P* = 0.02) and CON (*P* = 0.015) post‐cooling. No between‐group differences were seen for the other biomarkers. Mild to moderate chronic NFCI does not appear to be associated with a pro‐inflammatory state or oxidative stress. Baseline [IL‐10] and [syndecan‐1] and post‐heating [endothelin‐1] are the most promising candidates for diagnosing NFCI, but it is likely that a combination of tests will be required.

## Abstract (highlights)

- **What is the central question of this study?**
  Are biomarkers of endothelial function, oxidative stress and inflammation altered by non‐freezing cold injury (NFCI)?
- **What is the main finding and its importance?**
  Baseline plasma [interleukin‐10] and [syndecan‐1] were elevated in individuals with NFCI and cold‐exposed control participants. Increased [endothelin‐1] following thermal challenges might explain, in part, the increased pain/discomfort experienced with NFCI. Mild to moderate chronic NFCI does not appear to be associated with either oxidative stress or a pro‐inflammatory state. Baseline [interleukin‐10] and [syndecan‐1] and post‐heating [endothelin‐1] are the most promising candidates for diagnosis of NFCI.

## 1 INTRODUCTION

Prolonged exposure to cold (and often cold and wet) environments can cause non‐freezing cold injury (NFCI) in the hands and feet. The chronic symptoms include persistent pain, cold sensitivity, numbness and hyperhidrosis in variable combinations and severity (Golden et al., 2013; Kuht et al., 2019; Ungley et al., 1945). Historically, NFCI has been a major problem for the military; for example, 64% of the UK 3 Commando Brigade experienced symptoms of NFCI during the Falklands Conflict, although the conflict lasted only 25 days (Golden et al., 2013). The increased popularity of outdoor activities means that civilians are also at risk of NFCI (Longman et al., 2020; Oakley et al., 2022; Paal et al., 2013), as are those who are homeless (Parsons et al., 1993) or immobilized (Williams et al., 2005). Although the current phenotype is thought to be less severe than historical cases (Kuht et al., 2019), chronic symptoms can have a profound effect on an individual's quality of life and employability.

Currently, diagnosis of NFCI is largely based on clinical history and the symptoms presented. Recent studies investigating vascular function (Eglin et al., 2023) and neural function (Wright et al., 2023) in NFCI have advanced our understanding of the current phenotype, but have not highlighted a unique mechanism of injury or indicated a clinical test that could be used in the diagnosis and treatment of NFCI. Therefore, the aim of this study was to investigate whether a blood biomarker could be used in the diagnosis of chronic NFCI.

Plasma concentrations of various biomarkers have been shown to reflect endothelial dysfunction and disease severity in a variety of conditions (Kim et al., 2017; Kleinbongard et al., 2006; Strom et al., 2017) and might be more sensitive indicators of altered function. Nitric oxide (NO) is an important mediator of vasodilatation, and plasma [nitrite] is an established marker of endothelial NO production (Lauer et al., 2001), which might be impaired in individuals with cold sensitivity (Hope et al., 2014). Conversely, endothelin‐1 causes vasoconstriction and has been implicated in pain transmission (Smith et al., 2014). Given that NFCI is associated with slower rates of rewarming and greater discomfort after local cooling (Eglin et al., 2023), the roles of nitrite, nitrate and endothelin‐1 in NFCI were investigated.

It is not known whether the chronic phase of NFCI (after any hyperaemia following the injurious cold exposure has subsided but with persistent symptoms of cold sensitivity, pain or sensory impairment) is associated with inflammation. Pro‐inflammatory cytokines, such as interleukin‐6 (IL‐6) and tumour necrosis factor alpha (TNFα), have been associated with neuropathic pain (Leung & Cahill, 2010; Zhou et al., 2016), which has been reported in individuals with NFCI (Anand et al., 2017; Vale et al., 2017; Wright et al., 2023). Conversely, interleukin‐10 (IL‐10) is an anti‐inflammatory cytokine that is elevated in Raynaud's phenomenon, with disease progression being negatively correlated with [IL‐10] (Dziankowska‐Bartkowiak et al., 2004). Another marker of inflammation is E‐selectin, which is produced by endothelial cells and mediates the adhesion of neutrophils, monocytes and T lymphocytes to the vascular wall (Gorski et al., 2019). E‐Selectin is elevated in individuals with Raynaud's phenomenon who have abnormal nailfold capillaroscopic parameters (Gorski et al., 2019) and might predict progression to systemic sclerosis (Hebbar et al., 1995).

Reactive oxygen species (ROS) play a fundamental role in redox signalling and are produced endogenously as by‐products of normal cellular activity. When elevated to a degree that overwhelms the antioxidant defence systems, ROS production causes oxidative stress, which has been implicated in the aetiology and progression of many diseases through oxidative damage to proteins (e.g., protein carbonyl and nitrotyrosine), lipids [e.g., 4‐hydroxy‐2‐nonenal (4‐HNE)] and DNA. In an animal model (rat) of NFCI, ROS production was increased after cooling of the sciatic nerve (Geng et al., 2015). In addition, the cooling of blood vessels results in a rapid increase in ROS, which is attenuated by the antioxidant superoxide dismutase (SOD; Bailey et al., 2005; Das et al., 1991). Whether ROS are involved in the post‐hyperaemic phase of NFCI in humans is not known.

There are several markers of endothelial damage, of which von Willebrand factor (vWF), tissue type plasminogen activator (TTPA) and syndecan‐1 are the most relevant to NFCI. Von Willebrand factor is released in response to endothelial damage and mediates platelet aggregation and adhesion (Lip, 1997), with elevated levels of vWF being present in skin biopsies from individuals with NFCI (Anand et al., 2017). Tissue type plasminogen activator is also released from the endothelium and is elevated in Raynaud's phenomenon, with plasma [TTPA] being related to the severity of endothelial dysfunction (Gualtierotti et al., 2017; Marasini et al., 1992). Syndecans are endothelial glycocalyx proteins that convert endothelial shear stress into NO‐mediated vasodilatation (Kim et al., 2017). Elevated plasma [syndecan‐1] indicates degradation of the endothelial glycocalx, which has been associated with reduced flow‐mediated dilatation (Salmito et al., 2015).

Inflammation and ROS influence endothelial function independently and synergistically, with this cross‐talk being referred to as the vascular health triad (Ranadive et al., 2021). A battery of biomarkers that reflect inflammation, ROS and endothelial function might therefore provide better insight into the aetiology of NFCI than a single triad component. We therefore compared at baseline in individuals with chronic NFCI the plasma concentrations of biomarkers of endothelial function (nitrate, nitrite and endothelin‐1), inflammation (IL‐6, IL‐10, TNFα and E‐selectin), oxidative stress (protein carbonyl, 4‐HNE, SOD and nitrotyrosine) and endothelial damage (vWF, syndecan‐1 and TTPA) with those in cold‐exposed (COLD) and non‐cold‐exposed (CON) control participants. To determine whether physiological perturbations can highlight differences between groups, plasma [nitrate], [nitrite], [endothelin‐1], [IL‐6], [4‐HNE] and [TTPA] were also measured after whole‐body warming and foot cooling.

It was hypothesized that, compared with the control groups (COLD and CON), NFCI would show altered [biomarkers] indicative of endothelial dysfunction, inflammation and oxidative stress.

## 2 METHODS

The protocol received ethical approval from the Ministry of Defence Research Ethics Committee (909/MoDREC/18), and all participants gave written informed consent before undertaking any testing. The study complied with the *Declaration of Helsinki* (1964), as last revised at the 64th World Medical Association General Assembly, Brazil, 2013, except for registration in a database.

Testing was undertaken between January 2019 and October 2019, when the ambient outdoor temperature was 8.0 (4.8)°C. Given the need to recruit the NFCI group first, in order to enable matching of the control participants, the CON group were tested later, when the ambient temperature was higher [NFCI 5.3°C (range, −3 to 12°C); COLD 8.5 (range, 3–18°C); CON 11.2°C (range 6–18°C); *P* = 0.004 CON vs. NFCI]. The number of participants recruited was based on the sample size required for the vascular tests (Eglin et al., 2023); however, blood samples were not obtained from all of these participants at each time point.

The NFCI group were recruited from a regional UK military cold injuries clinic and had a current diagnosis of chronic NFCI. The NFCI diagnosis was based on a detailed case history of the circumstances (including duration of exposure, weather conditions, physical activity and clothing) of the initial injury; on subsequent and current symptoms; and on physical examination {including assessment of gait, balance, capillary refill and proprioception, standard blood tests (blood cell count, [cholesterol], [triglyceride] and [glycated haemoglobin]) and responses to pinprick, monofilament and vibration stimuli}. These tests were conducted by a medical doctor with >20 years of experience in reviewing NFCI cases and 7 years of running a regional NFCI clinic.

Cold‐exposed control (COLD) participants without a diagnosis of NFCI were recruited from UK Army soldiers and had therefore been exposed to similar winter military training exercises to the NFCI group. One member of the COLD group had a previous NFCI but was now considered, by the same medical doctor, to be completely recovered. The volunteers were selected to match the NFCI group for cold exposure, sex, race, age, aerobic fitness and body mass index as closely as possible.

Control (CON) participants with limited previous exposure to cold and no previous diagnosis of NFCI were recruited by word of mouth. The volunteers were selected to match the NFCI group for sex, race, age, aerobic fitness and body mass index as closely as possible. It was established through a verbal screening process during recruitment that CON participants did not partake in any sports/activities where they were likely to get cold (i.e., they took part in indoor sport/gym activities) and that they had not encountered any notable events of being cold where they might have sustained a cold injury. Descriptions of the cold exposure experienced by each group are detailed by Eglin et al. (2023).

Before arrival, participants were asked to refrain from smoking, caffeine ingestion and over‐the‐counter painkillers for 8 h. Participants attended the laboratory wearing T‐shirt and trousers. Participants were instructed not to undertake heavy exercise (more than their normal physical training session, which most participants were undertaking daily) and to refrain from alcohol consumption for 24 h. For 2 days before testing, participants were requested to avoid eating foods high in nitrate. Owing to logistical reasons, the time of day when the participants undertook the testing was not controlled. Five participants (one COLD and four CON) undertook all their testing in a single day. For the remaining participants, the baseline sample was collected on a different day from the post‐heating and post‐cooling samples (which were collected ∼90 min apart).

Venous blood samples from the antecubital vein were collected into EDTA and lithium heparin vacutainers after a 30 min resting period at 24°C (baseline) and immediately after a whole‐body heating challenge (post‐heating) and a foot cooling challenge (post‐cooling). For the whole‐body heating challenge, participants entered a chamber heated to [mean (SD)] 30.5 (0.9)°C, 37.4 (5.5)% relative humidity (Squirrel 2020; Grants Instruments, Cambridge, UK). After a seated rest period of 5 min, the participant undertook 12 min of cycling (Lode Corival CPET, Gronigen, The Netherlands) at 50 W to remove any central vasoconstrictor tone (Eglin et al., 2013). For the foot cooling challenge, participants were asked to remove their shoes and socks and sat in a room maintained at 25.1 (1.1)°C, 46.6 (8.4)% relative humidity. From the seated position, participants placed their feet on a water‐perfused aluminium footplate, which was maintained at 35°C. After a 10 min baseline period, the footplate was cooled at a rate of 1.7°C/min until it reached 15°C or until the participant reported discomfort. No differences in either toe skin blood flow or temperature were seen between groups during the foot cooling protocol (Eglin et al., 2023). All blood samples were placed in a cooled centrifuge (4°C) and spun at 4500*g* for 10 min. Plasma was aliquoted and initially frozen at −20°C until it could be transferred to a −80°C freezer (between 1 h and 4 days later) for subsequent analysis. [Nitrate] and [nitrite] at baseline, post‐heating and post‐cooling were analysed in duplicate from plasma collected in lithium heparin vacutainers using a NO analyser (Sievers NOA 280i; Analytix, Durham, UK), via a modification of the ozone chemiluminescence technique (Bateman et al., 2002).

All the other biomarkers were analysed by enzyme‐linked immunosorbent (ELISA) kits, with details of each kit provided in Table A1. Biomarkers were analysed following the instructions provided for each kit, and where necessary, technical support was sought from the manufacturer. The optical density of each sample well was measured using a Spectramax i3x microplate reader (Molecular Devices, USA). The optical densities of the standards (with known concentrations) were used to create standard curves (*y* = *mx* + *c*; where *y* is the optical density, *m* is the gradient of the line and *x* the concentration), from which concentrations of the samples were calculated. Initially, a dilution phase was undertaken with up to five samples to ensure that the sample concentration fell within the standard range of each kit (Table A1). Once established, all samples were analysed in duplicate and the mean and coefficient of variation (CV) calculated (Table A1).

At baseline, plasma [nitrate], [nitrite], [protein carbonyl], [4‐HNE], [endothelin‐1], [IL‐6], [IL‐10], [vWF], [syndecan‐1], [SOD], [TTPA], [TNFα], [E‐selectin] and [nitrotyrosine] were measured. [Nitrate], [nitrite], [4‐HNE], [endothelin‐1], [IL‐6] and [TTPA] were also measured post‐heating and post‐cooling. Analyses of [protein carbonyl], [syndecan‐1], [E‐selectin], [IL‐6] and [SOD] were undertaken in March 2020. Owing to the Covid‐19 pandemic, the other biomarkers were not analysed until later, between October 2020 and February 2021.

### 2.1 Data analyses

Data analyses were conducted on all samples. Samples that gave a negative expression value were considered as zero for analysis. The number of samples that lay 10% above or below the standard curve was noted, as was the number of samples that had a CV of >15% (Tables A1 and A2). Ideally, samples with a CV of >15% or which were >10% above the standard curve would have been removed from the analysis. However, given the small sample size, it was considered better to include these samples in the analysis, and this limitation is acknowledged in the Discussion.

In addition to absolute [biomarker] data, changes in plasma [nitrate], [nitrite], [4‐HNE], [endothelin‐1], [IL‐6] and [TTPA] from baseline after whole‐body heating and foot cooling were investigated between groups.

The balance between pro‐ and antioxidative and inflammatory markers was also investigated. The ratios of the antioxidant [SOD] to [IL‐6], [TNFα], [E‐selectin], [4‐HNE], [protein carbonyl], [TTPA], [vWF] and [syndecan‐1] at baseline were compared between NFCI, COLD and CON groups. The ratios of the anti‐inflammatory [IL‐10] to [IL‐6], [TNFα], [E‐selectin], [4‐HNE], [protein carbonyl], [TTPA], [vWF] and [syndecan‐1] at baseline were compared between NFCI and COLD groups, because IL‐10 was not detected in CON plasma samples. Nitrotyrosine was detected in only about half of the CON samples, two of the COLD samples and none of the NFCI samples; therefore, it was not included in this analysis. These biomarkers were chosen as comparators owing to their opposing roles compared with the other biomarkers (SOD, antioxidant; endothelin‐1, vasoconstrictor; and IL‐10, anti‐inflammatory).

The distribution of data was assessed using descriptive methods (skewness and kurtosis) and inferential statistics (Shapiro–Wilk test). When a normal distribution was violated, non‐parametric analyses were performed. Biomarkers measured at baseline only were analysed using a one‐way ANOVA or Kruskal–Wallis *H* test. Where appropriate, *post hoc* tests were conducted using Tukey's or Dunn's multiple comparisons test for parametric and non‐parametric data, respectively. [4‐HNE] and [IL‐6] at baseline, post‐heating and post‐cooling were analysed using a 3 × 3 (time × group) factorial ANOVA with *post hoc* pairwise comparisons. Baseline, post‐heating and post‐cooling [nitrate], [nitrite], [TTPA], [endothelin‐1] were analysed using a Kruskal–Wallis *H* test with Mann–Whitney *U* follow‐ups and a Friedman test with Wilcoxon *post hoc* tests. Changes in [biomarker] were analysed using a one‐way ANOVA or Kruskal–Wallis *H* test. Where appropriate, *post hoc* tests were conducted using Tukey's or Dunn's multiple comparisons test for parametric and non‐parametric data, respectively. The [SOD]:[biomarker] ratios and [endothelin‐1]:[nitrate] and [endothelin‐1]:[nitrite] ratios were analysed using a one‐way ANOVA, and where appropriate, Bonferroni *post hoc* tests were conducted. The [IL‐10]:[biomarker] ratios were analysed using Mann–Whitney *U* tests between NFCI and COLD.

Data are presented as the mean (SD) for parametric data and as the median [interquartile range (IQR)] for non‐parametric data unless otherwise stated. Statistical analysis was performed in SPSS v.25 (SPSS, Chicago, IL, USA) and GraphPad Prism v.9.3 (GraphPad Software, San Diego, CA, USA), and statistical difference and trends towards significance were accepted at *P* < 0.05 and *P* < 0.1, respectively.

## 3 RESULTS

### 3.1 Participants

Blood samples were collected from a total of 16 NFCI participants, six of whom had NFCI in their feet only, two in their hands only and eight in their hands and feet. The NFCI participants were seen 3.3 years (range, 2 months to 9 years and 5 months) after their initial injury, all of which occurred during military field exercises in freezing conditions (0 to −25°C). The aetiology of their injurious cold exposure and their current symptoms are detailed by Eglin et al. (2023).

Thirty‐two blood samples were collected at baseline (10 NFCI, 11 COLD and 11 CON), 38 post‐heating (13 NFCI, 13 COLD and 12 CON) and 30 post‐cooling (10 NFCI, 10 COLD and 10 CON). The demographics of the participants were similar and are shown in Table 1.

**TABLE 1:** Participant characteristics.

**Group**	***n***	**Age (years)**	**Height (cm)**	**Mass (kg)**	**Ethnicity**
NFCI	16 (2 F)	28.4 (4.3)	176.7 (6.3)	76.5 (6.7)	7 W; 7 Afr‐Car; 2 Mixed
COLD	17 (2 F)	31.5 (7.2)	176.7 (6.3)	84.1 (8.3)	9 W; 7 Afr‐Car; 1 Mixed
CON	14 (4 F)	25.7 (5.9)	176.4 (12.6)	76.9 (15.1)	7 W; 6 Afr‐Car; 1 Mixed

*Note*: The mean (SD) is presented for each group.

Abbreviations: Afr‐Car, African–Caribbean; COLD, cold‐exposed control participants; CON, non‐cold‐exposed control participants; F, female; Mixed, mixed race White–Caribbean; NFCI, individuals with chronic NFCI; W, White.

### 3.2 Endothelial function

**FIGURE 1:**
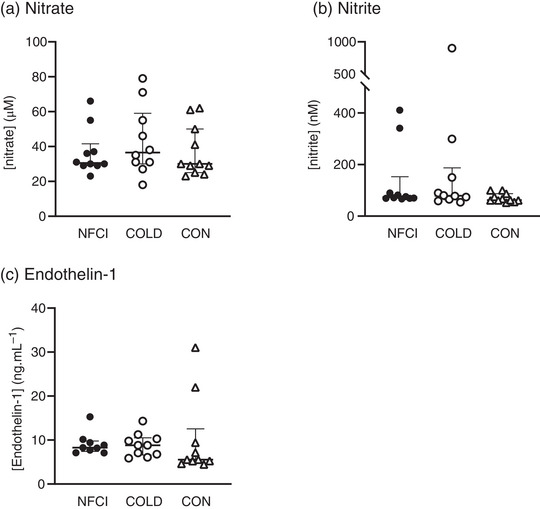
Endothelial function at baseline. Plasma concentrations of vasoactive biomarkers at baseline in the three groups: NFCI (individuals with chronic non‐freezing cold injury; filled circles, *n* = 10), COLD (cold‐exposed control participants; open circles, *n* = 11) and CON (non‐cold‐exposed control participants; open triangles, *n* = 11). Individual values and the median (interquartile range) are shown for nitrate (a), nitrite (b) and endothelin‐1 (c). No differences were observed between groups for [nitrate] (*P* = 0.478), [nitrite] (*P* = 0.169) or [endothelin‐1] (*P* = 0.198).

**FIGURE 2:**
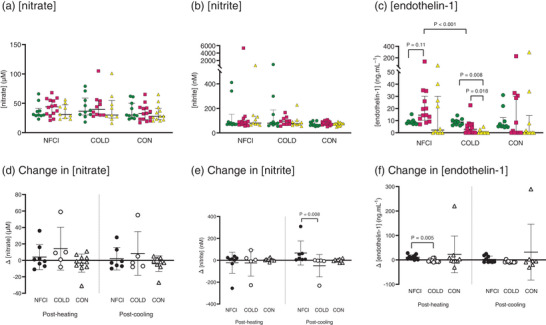
Endothelial function at baseline, post‐heating and post‐cooling. Plasma [nitrate] (a), [nitrite] (b) and [endothelin‐1] (c) at baseline (green circles), post‐heating (red squares) and post‐cooling (yellow triangles) and change in plasma [nitrate] (d), [nitrite] (e) and [endothelin‐1] (f) from baseline after whole‐body heating (Post‐heating) or foot cooling (Post‐cooling) in the three groups: NFCI (individuals with chronic non‐freezing cold injury), COLD (cold‐exposed control participants) and CON (non‐cold‐exposed control participants). Individual values and the median (interquartile range) are shown for absolute biomarker concentrations (a–c), and individual values and the mean (SD) are shown for the change in biomarker concentrations (d–f). Number of samples for [nitrate] and [nitrite] at baseline (NFCI = 10; COLD = 10; CON = 11), post‐heating (NFCI = 14; COLD = 10; CON = 14), post‐cooling (NFCI = 9; COLD = 9; CON = 12) and change in [nitrate] and [nitrite] post‐heating (NFCI = 8; COLD = 5; CON = 11) and post‐cooling (NFCI = 7; COLD = 5; CON = 9). Number of samples for [endothelin‐1] at baseline (NFCI = 9; COLD = 10; CON = 10), post‐heating (NFCI = 13; COLD = 13; CON = 12), post‐cooling (NFCI = 12; COLD = 10; CON = 10) and change in [endothelin‐1] post‐heating (NFCI = 10; COLD = 11; CON = 9) and post‐cooling (NFCI = 9; COLD = 10; CON = 7).

No differences were observed in plasma [nitrate] and [nitrite] between groups at baseline (*P* = 0.478 and *P* = 0.169), post‐heating (*P* = 0.138 and *P* = 0.390) and post‐cooling (*P* = 0.673 and *P* = 0.246) or within groups at the different time points (*P* = 0.124; Figures 1a,b and 2a,b).

Changes in plasma [nitrate] were similar between groups post‐heating (*P* = 0.155) and post‐cooling (*P* = 0.976; Figure 2d). The increase in [nitrite] post‐cooling in NFCI (median [IQR], 18 [55.0] nM, *n* = 7) was significantly greater than COLD (−4.0 [118.5], *n* = 5; *P* = 0.008) but not CON (−1.0 [25.5], *n* = 9; *P* = 0.067; Figure 2e). Change in [nitrite] was similar between groups post‐heating (*P* = 0.877; Figure 2e).

At baseline and post‐cooling, [endothelin‐1] was similar between groups (*P* = 0.198 and *P* = 0.249, respectively; Figure 1c). Post‐heating, [endothelin‐1] was greater in NFCI compared with COLD (*Z* = −3.776, *P* < 0.001; Figure 2c), and a trend towards it being greater than CON was observed (*Z* = −1.807, *P* = 0.071). In NFCI, [endothelin‐1] was elevated post‐heating compared with baseline (*Z* = −2.547, *P* = 0.011). In COLD, [endothelin‐1] was lower post‐cooling compared with both baseline (*Z* = 2.666, *P* = 0.008) and post‐heating (*Z* = 2.366, *P* = 0.018; Figure 2c).

A greater increase in [endothelin‐1] post‐heating was observed in NFCI compared with COLD (median [IQR], 9.2 [14.9] ng/ml, *n* = 10 vs. −5.3 [7.5] ng/ml, *n* = 11, *P* = 0.005; Figure 2f); however, no differences were observed between CON and either NFCI (*P* = 0.3135) or COLD (*P* = 0.4901). The change in [endothelin‐1] post‐cooling was similar between groups (*P* = 0.2729; Figure 2f).

### 3.3 Inflammation

**FIGURE 3:**
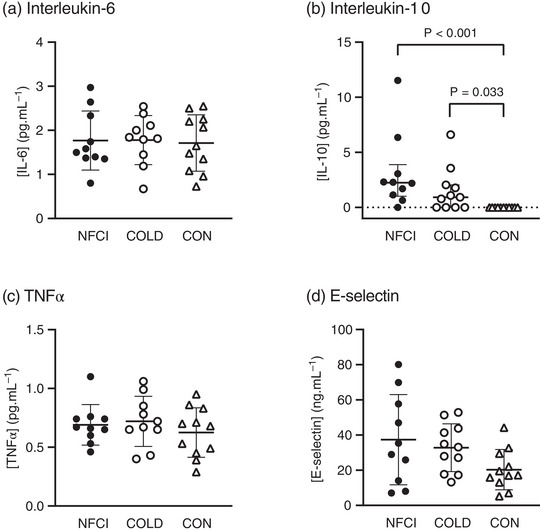
Inflammation at baseline. Plasma concentrations of inflammatory biomarkers at baseline in the three groups: NFCI (individuals with chronic non‐freezing cold injury; filled circles, *n* = 10), COLD (cold‐exposed control participants; open circles, *n* = 11) and CON (non‐cold‐exposed control participants; open triangles, *n* = 11). Individual values and the mean (SD) are shown for interleukin‐6 (a), the median (interquartile range) for interleukin‐10 (b) and the mean (SD) for tumour necrosis factor alpha (TNFα) (c) and E‐selectin (d). No differences were observed between groups for [interleukin‐6] (*P* = 0.935), [TNFα] (*P* = 0.545) or [E‐selectin] (*P* = 0.087).

**FIGURE 4:**
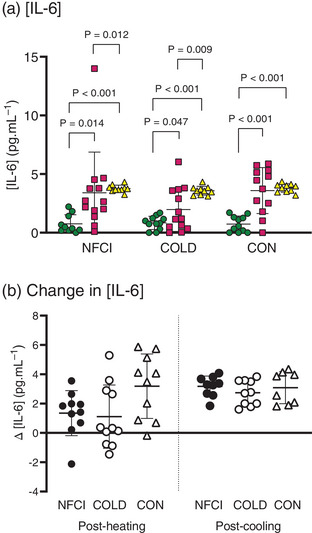
Concentrations of interleukin‐6 (IL‐6) at baseline, post‐heating and post‐cooling. (a) Plasma [IL‐6] at baseline (green circles), post‐heating (red circles) and post‐cooling (yellow triangles) and (b) change in plasma [IL‐6] from baseline after whole‐body heating (Post‐heating) or foot cooling (Post‐cooling) in the three groups: NFCI (individuals with chronic non‐freezing cold injury), COLD (cold‐exposed control participants) and CON (non‐cold‐exposed control participants). Individual values and the mean (SD) are shown. No differences between groups were observed between the change in [IL‐6] post‐heating (*P* = 0.052) or post‐cooling (*P* = 0.523). Number of samples for [IL‐6] at baseline, post‐heating and post‐cooling (NFCI = 9; COLD = 10; CON = 8) and change in [IL‐6] post‐heating (NFCI = 10; COLD = 11; CON = 10) and post‐cooling (NFCI = 9; COLD = 10; CON = 8).

There were no differences between groups in [IL‐6] at baseline, post‐heating or post‐cooling (time × group, *F* = 1.790, *P* = 0.176; group, *F* = 2.182, *P* = 0.135; Figures 3a and 4a). There was, however, an effect of time (*F* = 52.416, *P* < 0.001), with [IL‐6] being elevated post‐heating (NFCI, *P* = 0.014; COLD, *P* = 0.047; CON, *P* < 0.001) and post‐cooling (all groups, *P* < 0.001) compared with baseline (Figure 4a). In NFCI and COLD, [IL‐6] was also increased post‐cooling compared with post‐heating (*P* = 0.012 and *P* = 0.009, respectively; Figure 4a). Change in [IL‐6] was not different between groups either post‐heating (*P* = 0.052) or post‐cooling (*P* = 0.523).

Compared with CON, baseline plasma [IL‐10] was elevated in NFCI (*Z* = 3.951, *P* < 0.001; Figure 3b) and COLD (*Z* = 2.538, *P* = 0.033, Figure 3b); however, no difference was observed between COLD and NFCI (*P* = 0.422; Figure 3b).

No significant differences between groups were observed for baseline plasma [TNFα] (*F* = 0.621, *P* = 0.545; Figure 3c) or [E‐selectin] (*F* = 2.655, *P* = 0.087, Figure 3d).

### 3.4 Oxidative stress

**FIGURE 5:**
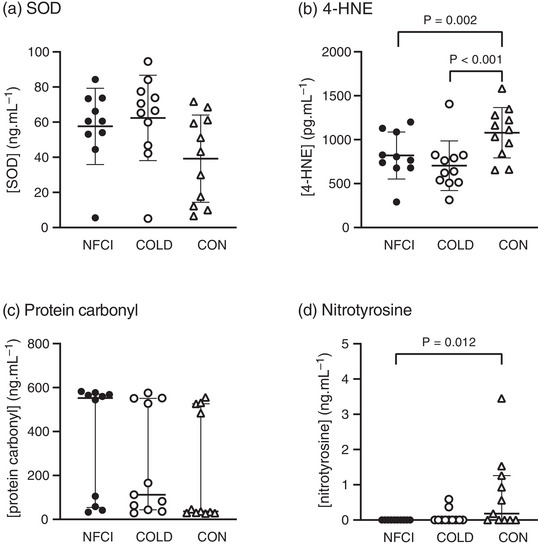
Oxidative stress. Plasma concentrations of oxidative stress biomarkers at baseline in the three groups: NFCI (individuals with chronic non‐freezing cold injury; filled circles, *n* = 10), COLD (cold‐exposed control participants; open circles, *n* = 11) and CON (non‐cold‐exposed control participants; open triangles, *n* = 11). Individual values and the mean (SD) are shown for superoxide dismutase (SOD; a); 4‐hydroxy‐2‐noneal (4‐HNE; b) and the median (interquartile range) for protein carbonyl (c) and nitrotyrosine (d). No differences were observed between groups for [SOD] (*P* = 0.071) or [protein carbonyl] (*P* = 0.052).

No difference in plasma [SOD] (*P* = 0.071; Figure 5a) or [protein carbonyl] (*P* = 0.052; Figure 5c) was observed between groups at baseline. Nitrotyrosine was not detected in any samples from the NFCI group and only detected in two of 11 of the COLD group and six of 11 in the CON group. Therefore, the significantly higher [nitrotyrosine] in CON compared with NFCI (*Z* = 2.887, *P* = 0.012; Figure 5d) should be viewed with caution. No differences in [nitrotyrosine] were observed between NFCI and COLD (*P* > 0.999) or between COLD and CON (*P* = 0.109).

**FIGURE 6:**
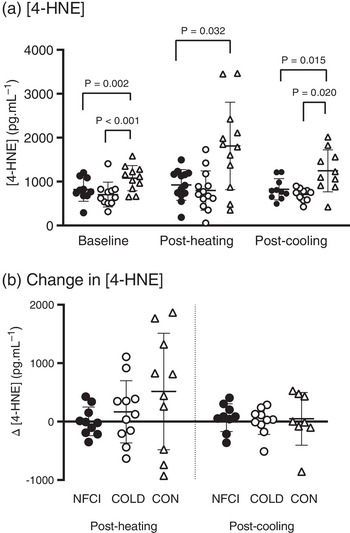
Concentrations of 4‐hydroxy‐2‐noneal (4‐HNE) at baseline, post‐heating and post‐cooling. (a) Plasma [4‐HNE] at baseline, post‐heating and post‐cooling and (b) changes in [4‐HNE] from baseline at post‐heating and post‐cooling in the three groups: NFCI (individuals with chronic non‐freezing cold injury; filled circles), COLD (cold‐exposed control participants; open circles) and CON (non‐cold‐exposed control participants; open triangles). Individual values and the mean (SD) are shown. No differences between groups were observed for the change in [4‐HNE] post‐heating (*P* = 0.229) or post‐cooling (*P* = 0.914). Number of samples for [4‐HNE] at baseline, post‐heating and post‐cooling (NFCI = 9; COLD = 10; CON = 8) and change in [4‐HNE] post‐heating (NFCI = 10; COLD = 11; CON = 10) and post‐cooling (NFCI = 9; COLD = 10; CON = 8).

Compared with CON, [4‐HNE] was lower in NFCI at baseline (*P* = 0.002; Figure 5b), post‐heating (*P* = 0.032; Figure 6a) and post‐cooling (*P* = 0.015; Figure 6a). The COLD participants also had reduced plasma [4‐HNE] compared with CON at baseline (*P* < 0.001; Figure 5b) and post‐cooling (*P* = 0.020; Figure 6a) but not post‐heating (*P* = 0.064). No significant differences in plasma [4‐HNE] were observed between NFCI and COLD (baseline, *P* = 0.501; post‐heating, *P* = 0.692; post‐cooling, *P* = 0.364). Within groups, there were no significant differences in [4‐HNE] between baseline, post‐heating and post‐cooling samples (*P* = 0.265). Changes in plasma [4‐HNE] from baseline to post‐heating (*P* = 0.229) and post‐cooling (*P* = 0.914) were similar between groups (Figure 6b).

### 3.5 Endothelial damage

**FIGURE 7:**
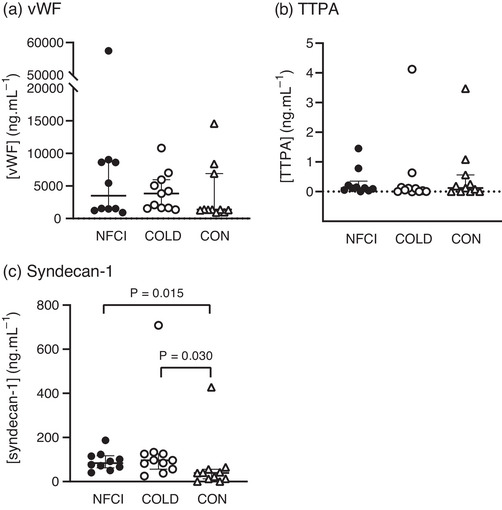
Endothelial damage. Plasma concentrations of biomarkers indicating endothelial damage at baseline in the three groups: NFCI (individuals with chronic non‐freezing cold injury; filled circles, *n* = 10), COLD (cold‐exposed control participants; open circles, *n* = 11) and CON (non‐cold‐exposed control participants; open triangles, *n* = 11). Individual values and the median (interquartile range) are shown for von Willebrand Factor (vWF; a); tissue type plasminogen activator (TTPA; b) and syndecan‐1 (c). No differences were observed between groups for [vWF] (*P* = 0.166) or [TTPA] (*P* = 0.522).

At baseline, no differences in plasma concentrations of vWF were observed between groups (*P* = 0.166; Figure 7a).

**FIGURE 8:**
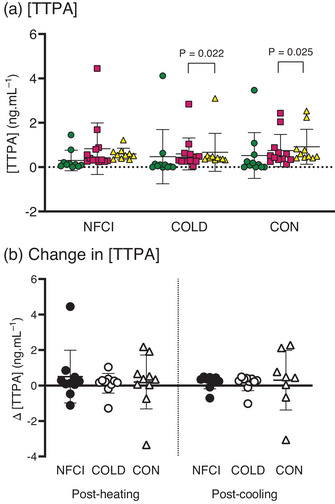
Concentrations of tissue type plasminogen activator (TTPA) at baseline, post‐heating and post‐cooling. (a) Plasma [TTPA] at baseline (green circles), post‐heating (red squares) and post‐cooling (yellow triangles) and (b) change in plasma [TTPA] from baseline after whole‐body heating (Post‐heating) or foot cooling (Post‐cooling) in the three groups: NFCI (individuals with chronic non‐freezing cold injury), COLD (cold‐exposed control participants) and CON (non cold‐exposed control participants). Individual values and the mean (SD) are shown. No differences between groups were observed for the change in [TTPA] post‐heating (*P* = 0.789) or post‐cooling (*P* = 0.567). Number of samples for [TTPA] at baseline (NFCI = 10; COLD = 11; CON = 11), post‐heating (NFCI = 13; COLD = 13; CON = 12), post‐cooling (NFCI = 9; COLD = 10; CON = 10) and change in [TTPA] post‐heating (NFCI = 10; COLD = 11; CON = 10) and post‐cooling (NFCI = 9; COLD = 10; CON = 8).

Plasma concentrations of TTPA were similar between groups at baseline (*P* = 0.522), post‐heating (*P* = 0.606) and post‐cooling (*P* = 0.085; Figures 7b and 8a), with no between‐group differences observed for changes in [TTPA] from baseline to post‐heating or post‐cooling (*P* = 0.789 and *P* = 0.567, respectively; Figure 8b). A significant difference across time points was observed (χ^2^ = 17.85, *P* < 0.001), with [TTPA] being elevated post‐heating (*Z* = −2.224, *P* = 0.026) and post‐cooling (*Z* = −2.163, *P* = 0.031) compared with baseline, and with post‐heating [TTPA] being greater than post‐cooling (*Z* = −2.391, *P* = 0.017). However, when separated by groups, there was no significant difference between time points in the NFCI group (*P* = 0.097). In the COLD and CON groups, [TTPA] post‐cooling was significantly greater only than post‐heating (*Z* = −2.293, *P* = 0.022 and *Z* = −2.245, *P* = 0.025, respectively). In COLD participants, [TTPA] was numerically greater post‐cooling compared with baseline but did not reach statistical significance (*Z* = −1.684, *P* = 0.092, *r* = 0.37, medium effect size).

Plasma [syndecan‐1] at baseline was elevated in NFCI and COLD compared with CON (median [IQR], 84.0 [124.6] and 96.3 [69.2] vs. 38.7 [43.3] ng/ml; *P* = 0.015 and *P* = 0.030, respectively; Figure 7c). No difference in [syndecan‐1] was observed between NFCI and COLD (*P* > 0.999).

### 3.6 Biomarker ratio analysis

The ratio of [SOD]:[4‐HNE] was greater in COLD (mean [SD], 0.107 [0.057], *n* = 11) compared with CON (0.043 [0.033], *n* = 11, *P* = 0.014; Table 2). The ratio of [SOD] to all the other biomarkers was similar between groups (Table 2).

**TABLE 2:** Ratio of [SOD] and [IL‐10] to various plasma [biomarkers].

	**Compared with SOD**					**Compared with IL‐10**			
				**ANOVA**				**Mann–Whitney *U*‐test**	
**Biomarker**	**NFCI** **(*n* = 10)**	**COLD** **(*n* = 11)**	**CON** **(*n* = 11)**	***F***	***P*‐value**	**NFCI** **(*n* = 9)**	**COLD** **(*n* = 7)**	***Z***	***P*‐value**
IL‐6^a^	169.1 (154.1)	91.5 (82.2)	55.1 (54.5)	2.537	0.101	8.74 (9.69)	2.66 (2.10)	−1.969	**0.049**
TNFα^b^	85.4 (38.1)	85.0 (35.1)	65.7 (45.5)	0.836	0.444	4.49 (3.36)	2.94 (2.86)	−1.532	0.126
E‐selectin	2.29 (1.89)	1.98 (0.82)	1.97 (1.10)	0.198	0.822	0.10 (0.09)	0.06 (0.05)	−1.014	0.311
4‐HNE	0.08 (0.05)	0.11 (0.06)	0.04 (0.03)	4.823	**0.016**	0.01 (0.01)	0.01 (0.01)	−0.212	0.832
Protein carbonyl	0.61 (0.76)	0.68 (0.74)	0.69 (0.75)	0.190	0.967	0.02 (0.02)	0.03 (0.04)	−0.265	0.791
TTPA^c^	644 (615)	758 (873)	198 (218)	1.587	0.229	35.8 (42.7)	13.4 (13.5)	−0.867	0.386
vWF	0.03 (0.03)	0.02 (0.02)	0.03 (0.03)	0.000	0.883	0.00 (0.00)	0.00 (0.00)	−0.477	0.633
Syndecan‐1^d^	0.74 (0.48)	0.74 (0.58)	1.21 (1.09)	1.296	0.290	0.04 (0.03)	0.02 (0.01)	−1.535	0.125

*Note*: Values are the mean (SD) ratio of the plasma concentrations of SOD and IL‐10 to other biomarker concentrations at baseline in NFCI (individuals with chronic NFCI), COLD (cold‐exposed control participants) and CON (non‐cold‐exposed control participants) groups. ANOVA statistics are given for the between‐group comparison for [SOD] ratios, and Mann–Whitney *U* statistics are given for the comparison between NFCI and COLD for [IL‐10] ratios; IL‐10 was not detected at baseline in the CON group.

^a^ *n* for [SOD]:[IL‐6]: NFCI = 9, COLD = 9 and CON = 8; and for [IL‐10]:[IL‐6]: NFCI = 8 and COLD = 9.

^b^ *n* for [IL‐10]:[TFNα]: NFCI = 9 and COLD = 6.

^c^ *n* for [SOD]:[TTPA]: NFCI = 9, COLD = 7 and CON = 7; and for [IL‐10]:[TTPA]: NFCI = 9 and COLD = 5.

^d^ *n* for [SOD]:[syndecan‐1]: NFCI = 10, COLD = 11 and CON = 9.

Abbreviations: 4‐HNE, 4‐hydroxy‐2‐noneal; IL‐6, interleukin‐6; IL‐10, interleukin‐10; SOD, superoxide dismutase; TNFα, tumour necrosis factor alpha; TTPA, tissue type plasminogen activator; vWF, von Willebrand factor.

The ratio of [IL‐10]:[IL‐6] was greater in NFCI (median [IQR], 6.26 [5.10], *n* = 8) compared with COLD (1.14 [2.74], *n* = 4, *Z* = −1.969, *P* = 0.049, Table 2). The ratio of [IL‐10] to all the other biomarkers was similar in NFCI and COLD groups (Table 2).

The ratio of [endothelin‐1] to [nitrate] and [nitrite] was similar between groups (Table 3).

**TABLE 3:** Ratio of [endothelin‐1] to [nitrate] and [nitrite].

	**NFCI**	**COLD**	**CON**		
	**(*n* = 8)**	**(*n* = 8)**	**(*n* = 10)**	***F***	***P*‐value**
Nitrate	0.290 (0.122)	0.276 (0.139)	0.337 (0.401)	0.128	0.880
Nitrite	0.114 (0.055)	0.110 (0.067)	0.137 (0.124)	0.237	0.790

*Note*: Values are the mean (SD) ratio of plasma plasma [endothelin‐1] to [nitrate] and [nitrite] at baseline in NFCI (individuals with chronic NFCI), COLD (cold‐exposed control participants) and CON (non‐cold‐exposed control participants) groups. ANOVA statistics are given for the between‐group comparisons.

## 4 DISCUSSION

This is the first study to contain a comprehensive investigation of biomarkers associated with inflammation, oxidative stress, endothelial function and damage in individuals with chronic NFCI and matched control participants. The principal new findings are that inflammation, oxidative stress and alterations in plasma [endothelin‐1], [nitrite] and [nitrate] at baseline are not involved in chronic NFCI. Elevations in [endothelin‐1] following thermal challenges, however, might help to explain the increased pain/discomfort experienced with NFCI. Oxidative stress does not appear to be present in chronic NFCI, because plasma [nitrotyrosine], [protein carbonyl] and [4‐HNE] were not elevated compared with the control groups and, conversely, 4‐HNE was lower in NFCI. In addition, the antioxidant [SOD] was not reduced in chronic NFCI, nor were there any differences in the redox balance between NFCI and the control groups. Damage to the endothelial glycocalyx might occur as a result of cold exposure, as suggested by the fact that plasma [syndecan‐1] was increased in both NFCI and COLD. However, other markers of endothelial damage (plasma [vWF] and [TTPA]) were similar between groups. The results from this study indicate that baseline circulating concentrations of IL‐10 and syndecan‐1, in addition to post‐heating concentrations of endothelin‐1, are the most promising candidates for chronic NFCI diagnosis. As such, the hypothesis that levels of biomarkers associated with endothelial dysfunction, inflammation and oxidative stress would differ between NFCI and control participants cannot be accepted clearly for the cohort examined. The differences observed were limited or were potentially attributable to non‐detection of a biomarker or outliers or included the NFCI and COLD groups. In clinical terms, a biomarker that can predict the presence of NFCI would be of great value in its diagnosis and treatment. However, to be relevant clinically, this biomarker or combination of biomarkers would also need to show specificity for NFCI, to have prognostic value or to be correlated with NFCI severity (Frijhoff et al., 2015).

The plasma concentrations of the 14 biomarkers from the three groups are discussed below with reference to the results of the neurological and vascular tests (conducted in the same participants) reported in the preceding two papers (Eglin et al., 2023; Wright et al., 2023).

### 4.1 Endothelial function

Nitric oxide is a potent vasodilator. Increasing NO bioavailability with organic nitrates (via a glyceryl trinitrate spray) can improve skin blood flow in individuals with cold sensitivity (Hope et al., 2014) and Raynaud's phenomenon (Anderson et al., 2002), and inorganic nitrate can improve skin blood flow in individuals with Raynaud's phenomenon (Shepherd et al., 2019). Nitrate (NO_3_^−^) can be reduced to nitrite (NO_2_^−^), which can be reduced further to NO in a reversible process. Circulating nitrite is also reflective of NO derived from endothelial NO synthase (Kleinbongard et al., 2006), and thus increases in [nitrite] occur after NO synthase‐evoked vasodilatation (Rassaf et al., 2006). Elevation in circulating plasma [nitrite] increases the potential for NO generation independently of endothelial NO synthase activation, which is attenuated in conditions of oxidative stress (Li & Förstermann, 2013). Lowered plasma [nitrate] is associated with endothelial dysfunction and cardiovascular disease (Kleinbongard et al., 2006; Zhang et al., 2016), with [nitrate] being positively correlated with flow‐mediated dilatation (Kleinbongard et al., 2006) and negatively correlated with cardiovascular risk factors (Kleinbongard et al., 2006) and disease severity (Zhang et al., 2016). However, no differences in [nitrite] were observed between groups at any time point (Figures 1 and 2), indicating that any endothelial dysfunction is mild compared with cardiovascular disease, in which [nitrite] is increased (Kleinbongard et al., 2006). Indeed, plasma [nitrite] in NFCI, COLD and CON was similar to that reported in individuals with cold sensitivity (74 ± 49 nM; Eglin et al., 2017) and Raynaud's phonomenon (69 ± 23 nM; Shepherd et al., 2019) using an identical analysis technique. It was anticipated that whole‐body heating, which induces cutaneous vasodilatation, would increase [nitrite], but no differences were observed between baseline and post‐heating in any of the groups. It could be that the increase in [nitrite] is transient, occurring at the onset of vasodilatation. This is supported by Rassaf et al. (2006), who measured increases in [nitrite] at 10 s intervals during flow‐mediated dilatation of the forearm and showed that peak plasma [nitrite] coincided with peak brachial artery diameter after 60 s. Interestingly, NFCI showed an increase in [nitrite] post‐cooling compared with baseline, whereas the control groups showed a decrease or no change (Figure 2e). Why [nitrite] was elevated post‐cooling in the NFCI group is unclear, since the groups had similar reductions in cutaneous vascular conductance and skin temperature at the end of the foot cooling protocol (Eglin et al., 2023).

Increased levels of endothelin‐1 have been reported in individuals with Raynaud's phenomenon (Gorski et al., 2019; Mangiafico et al., 1996; Zamora et al., 1990), but no differences in [endothelin‐1] were observed between groups at baseline (Figure 1c). However, post‐heating, [endothelin‐1] and change in [endothelin‐1] were greater in NFCI compared to COLD (Figure 2c,f). The elevated [endothelin‐1] in NFCI might explain their cooler toe skin temperature during the cold sensitivity test, which followed immediately after the post‐heating blood sample was taken (see Eglin et al., 2023). However, despite an elevated [endothelin‐1] in NFCI compared with COLD participants post‐heating and post‐cooling (Figure 2c), no differences in toe skin blood flow during the cold sensitivity test or the foot cooling protocol were observed between these groups. Using post‐heating [endothelin‐1] in the diagnosis of NFCI might be practical only if conducted as part of a cold sensitivity test, as in the present study.

Endothelin‐1 has been implicated in pain transmission by directly acting on endothelin receptors on the peripheral endings of nociceptors and by potentiating the effects of other algogens (Smith et al., 2014). Intradermal injection of endothelin causes pain, cold hyperalgesia and reduced warm detection but not cold detection (Hans et al., 2007). Therefore, the increased [endothelin‐1] in the NFCI group might have contributed to their increased reports of cold, discomfort and pain during foot cooling (cold sensitivity test and foot cooling protocol) compared with the control groups (see Eglin et al., 2023).

The balance between biomarkers implicated in vasoconstriction and vasodilatation did not appear to be altered by NFCI, because the ratio of [endothelin‐1] to both [nitrate] and [nitrite] was similar between groups (Table 2). This is supported by the findings of the vascular tests, in which baseline cutaneous vascular conductance was similar between groups. However, it should be borne in mind that there are many other mediators of vasoconstriction and vasodilatation that might be altered with chronic NFCI but that were not measured in the present study.

### 4.2 Inflammation

Interleukin‐6 is an inflammatory cytokine that is expressed immediately and transiently in response to stressors and appears to play an important role in the pathogenesis of neuropathic pain (Zhou et al., 2016). In animal models of pain, elevated levels of IL‐6 have been found in the spinal cord and dorsal root ganglia, and treatment with anti‐IL‐6 antibodies has reduced mechanical allodynia and thermal hyperalgesia associated with pathological pain (Zhou et al., 2016). Plasma [IL‐6] was not elevated in NFCI compared with either control group at baseline, post‐heating or post‐cooling (Figures 3a and 4). These results might reflect the fact that NFCI demonstrated hyposensitivity to mechanical stimuli (mechanical detection threshold: NFCI, 20.3 [30.6] nm; COLD, 10.3 [5.6] nm; CON, 4.0 [3.4] nm, *P* = 0.001) and detection of warmth (warm detection threshold: NFCI, 45.9 [4.7]°C; COLD, 43.4 [2.7]°C; CON, 43.5 [4.7]°C, *P* = 0.040) but not mechanical allodynia or thermal hyperalgesia in the foot (Wright et al., 2023). In contrast, plasma [IL‐6] is elevated in individuals with Raynaud's phenomenon compared with control participants (Gualtierotti et al., 2017), indicating that the pathophysiology of Raynaud's phenomenon and chronic NFCI differ.

Plasma [IL‐6] was increased in response to whole‐body heating when compared with baseline levels in all groups (Figure 4). Similar increases in [IL‐6] have been reported after exercise or exposure to heat and might be related to fatigue (Costello et al., 2018; Robson‐Ansley et al., 2009). As far as we are aware, this is the first study to show an increase in [IL‐6] after foot cooling (Figure 4). Watkins et al. (2018) reported no changes in plasma [IL‐6] after 15 min of wearing a cooling vest or immersion of the forearms in 16°C water or consumption of ice slurry. Given that these cold stimuli are greater than the foot cooling in the present study, the elevated [IL‐6] post‐cooling could reflect a continued rise in [IL‐6] after the whole‐body heating (the post‐cooling blood sample was taken ∼90 min after the whole‐body heating).

Tumour necrosis factor alpha is a pro‐inflammatory cytokine and is associated with neuropathic pain (Leung & Cahill, 2010) and cardiovascular diseases (Cimminiello et al., 1994). Baseline plasma [TNFα] was similar in NFCI and the control groups (Figure 3c; Table 3) and corresponds to the findings in Raynaud's phenomenon (Cimminiello et al., 1994). In contrast to IL‐6 and TNFα, which are pro‐inflammatory cytokines, IL‐10 is anti‐inflammatory and plays an important role in preventing inflammatory and autoimmune pathologies (Iyer & Cheng, 2012). Interleukin‐10 is elevated in Raynaud's phenomenon, with the duration/progression of the disease being negatively correlated with [IL‐10] (Dziankowska‐Bartkowiak et al., 2004). Elevations in [IL‐10] have also been reported in Raynaud's phenomenon after inorganic nitrate supplementation (Shepherd et al., 2019). Given that elevated [IL‐10] can inhibit pro‐inflammatory cytokine production (Iyer & Cheng, 2012), the increased [IL‐10] in NFCI and COLD compared with CON at baseline (Figure 3b) might explain why [IL‐6] and [TNFα] were similar between groups (Figure 3a,c).

E‐Selectin is produced by endothelial cells and mediates the adhesion of neutrophils, monocytes and T lymphocytes to the vascular wall in the early stage of endothelial activation. E‐Selectin is elevated in individuals with Raynaud's phenomenon, which has been related to abnormal nailfold capillaroscopic parameters (Gorski et al., 2019) and might predict progression to systemic sclerosis (Hebbar et al., 1995). In the present study, no differences in plasma [E‐selectin] were observed between groups (Figure 3d).

From these results, it would appear that chronic NFCI is not associated with inflammation, as baseline levels of IL‐6, TNFα and E‐selectin were similar to the control groups. In contrast, the ratio between [IL‐10] and [IL‐6] indicated that the balance was towards anti‐inflammatory in the NFCI group (Table 2). This does not preclude the possibility that inflammation might be present in the acute phases of NFCI or in more severe cases. The elevated levels of IL‐10 at baseline (Figure 3b) could either be suppressing IL‐6 and TNFα release or could be the result of upregulation by other inflammatory cytokines (Iyer & Cheng, 2012) that were not measured in the present study.

### 4.3 Oxidative stress

4‐Hydroxynonenal is a product of lipid oxidation and, at low levels, is important for redox signalling and might trigger an antioxidant response. Elevated levels of 4‐HNE have been implicated in the pathology of many diseases (Breitzig et al., 2016), thus 4‐HNE is a marker of oxidative stress and, potentially, of disease progression (Breitzig et al., 2016). In the present study, plasma [4‐HNE] was lower in NFCI compared with CON at baseline, post‐heating and post‐cooling (Figures 5b and 6). The elevated [4‐HNE] at all time points in CON compared with NFCI and COLD appeared to be a consequence of an elevated [4‐HNE] at baseline, because no differences between groups were observed when looking at changes in [4‐HNE]. Plasma [4‐HNE] in each group was considerably lower than the estimated normal range (Dalleau et al., 2013) and that measured in mountaineers (Siervo et al., 2014).

Nitric oxide and superoxide can produce perioxynitrite, which is a potent oxidant with a half‐life of <1 s. Perioxynitrite, in turn, can react with tyrosine to produce nitrotyrosine, which is more stable and therefore a suitable marker for NO‐mediated tissue damage (Herce‐Pagliai et al., 1998). Nitrotyrosine was not detected in any of the plasma samples from NFCI, whereas it was detected in about half of the CON participants (Figure 5d). Likewise, individuals with primary Raynaud's phenomenon were found to have lower levels of plasma [nitrotyrosine] than control participants (Kingdon et al., 2006). Given that nitrite/nitrate concentrations were similar between groups (Figures 2 and 3), the lower [nitrotyrosine] in NFCI might be a result of upregulated degradation of nitrated proteins (Kingdon et al., 2006). Protein carbonyl, another marker of oxidative stress, was similar between groups (Figure 5c). However, given that many of the sample concentrations were above the standard curve (Table A1), these results should be interpreted with caution.

Superoxide dismutase protects cells against the damaging effect of ROS by catalysing the dismutation of superoxide anion free radicals into molecular oxygen and hydrogen peroxide (Younus, 2018). Reactive oxygen species are involved in the endothelial and tissue damage associated with ischaemia and reperfusion, and SOD appears to be important in protecting against this damage (Bertuglia & Giusti, 2003; Yang et al., 1994). Superoxide dismutase levels are reduced in individuals with diabetic sensorimotor polyneuropathy (Strom et al., 2017) and Raynaud's phenomenon (Balbir‐Gurman et al., 2007). No difference in baseline [SOD] was seen between the NFCI and control groups (Figure 5a), suggesting that antioxidant defence was not compromised in chronic NFCI. In addition, the redox balance was similar in the NFCI and control groups (as assessed from the ratio of [SOD]:[protein carbonyl] at baseline; Table 2). Although animal models of NFCI indicate a role for ROS in the tissue damage immediately after cooling (Das et al., 1991; Geng et al., 2015), the results from the present study indicate that chronic NFCI is not associated with oxidative stress (based on the biomarkers measured). Although every effort was made to recruit individuals with severe chronic NFCI, they probably had a less severe phenotype than those referred to neuropathic pain clinics (Anand et al., 2017; Vale et al., 2017) and the cases originally described by Ungley in 1945 (Kuht et al., 2019; Ungley et al., 1945). Therefore, it is possible that more severe cases of NFCI will be associated with oxidative stress and, as with other disorders, the magnitude of oxidative stress could be a marker of disease severity (Breitzig et al., 2016). There are also other biomarkers of oxidative stress that we did not measure in the present study that might be more sensitive and reveal differences between NFCI and the control groups.

### 4.4 Endothelial damage

Von Willebrand factor is stored in endothelial cells and is released in response to endothelial damage and has therefore been proposed as a marker of endothelial dysfunction (Lip, 1997). Calf skin biopsies taken from individuals with chronic NFCI were found to have increased levels of vWF (Anand et al., 2017), and individuals with Raynaud's phenomenon showed elevated plasma [vWF] (Gualtierotti et al., 2017). In the present study, plasma [vWF] was not elevated in NFCI compared with the control groups (Figure 7a), indicating that endothelial function was similar between groups. This supports the results of the vascular tests (post‐occlusive reactive hyperaemia, cutaneous local heating and iontophoresis of acetylcholine), in which no differences in responses between groups were observed (Eglin et al., 2023). However, plasma [vWF] varies fivefold in the healthy population, with genetics accounting for ≤75% of the variation (Desch et al., 2013); therefore, this wide variation might mask any small group differences.

Tissue type plasminogen activator is released from the endothelium and converts plasminogen to plasmin, which degrades fibrin clots (Muldowney & Vaughan, 2002). Plasma [TTPA] is elevated in Raynaud's phenomenon and is related to the severity of endothelial dysfunction (Gualtierotti et al., 2017; Marasini et al., 1992). Although plasma [TTPA] was elevated after foot cooling (Figure 8), there was no difference between groups. Vasoconstriction during the cooling might have caused the increase in [TTPA], because ischaemia is known to increase TTPA levels within the brain (Yepes, 2015).

Syndecans are transmembrane core proteins within the endothelial glycocalyx that maintain endothelial integrity and convert cytoskeletal sheer stress in the endothelial cells to NO‐mediated vasodilatation (Kim et al., 2017). Individuals with nephrotic syndrome have reduced flow‐mediated dilatation, which has been associated with elevated levels of syndecan‐1, suggesting that the endothelial dysfunction in these individuals involved glycocalyx damage (Salmito et al., 2015). Cold exposure might be associated with endothelial damage, as levels of syndecan‐1 were found to be higher in NFCI and COLD compared with CON (Figure 7c); however, this was not supported by the plasma vWF and TTPA concentrations or the results of the vascular tests (Eglin et al., 2023).

### 4.5 Limitations

There are a number of limitations to this study. Blood samples were not collected from all the participants for logistical reasons (particularly for baseline samples when vascular or neural tests were also being conducted) or the participant declining to have a blood sample taken, resulting in small sample sizes. Therefore, all samples were included in the analysis, although some of them fell outside desirable boundaries, with IL‐10, E‐selectin and vWF being most affected (Tables A2 and A3). Although the plasma samples were stored for ≤2 years at −80°C, it is not thought that this would have affected the results (Spies‐Martin et al., 2002), especially given that samples from the NFCI, COLD and CON groups were analysed at the same time. It is considered that a robust, clear and clinically applicable marker of NFCI should still have been able to emerge despite these limitations.

There was considerable individual variability in [biomarker] and in the vascular and neural responses (Eglin et al., 2023; Wright et al., 2023) in each of the groups; such variability in response has been highlighted previously (Tipton et al., 2020). There might have been some overlap between the NFCI and COLD groups; it became apparent during testing and discussion with the volunteers that some of the COLD group might have had undiagnosed NFCI (they had not gone to the medics for fear of the consequences for their careers). It seems unlikely that some of the NFCI group contained individuals without NFCI, given how carefully the NFCI group of volunteers were selected. This might therefore have masked any differences in plasma biomarker concentrations between these two groups.

Given the relatively mild phenotype of chronic NFCI in our participants compared with other studies (Anand et al., 2017; Golden et al., 2013; Ungley et al., 1945; Vale et al., 2017), it cannot be concluded that oxidative stress, inflammation and endothelial dysfunction are not present in more severe cases of chronic NFCI or in earlier phases of the condition. However, the participants in our study are probably more representative of the current NFCI phenotype (Kuht et al., 2019). Additionally, other biomarkers not analysed in the present study might have demonstrated differences between the NFCI cases and control participants.

## 5 CONCLUSION

It is concluded that neither inflammation, oxidative stress nor alterations in the vasoactive biomarkers endothelin‐1, nitrite and nitrate at baseline are involved in the chronic phase of NFCI. Damage to the endothelial glycocalyx might occur as a result of cold exposure, as plasma [syndecan‐1] was increased in both NFCI and COLD. However, other measures of endothelial function (post‐occlusive reactive hyperaemia, cutaneous local heating, iontophoresis of acetylcholine, plasma vWF and TTPA concentrations) were similar between groups. Of the 14 biomarkers measured in the present study, baseline IL‐10 and syndecan‐1 and post‐heating endothelin‐1 are the most promising candidates as clinical biomarkers for chronic NFCI and should be investigated in a longitudinal study of NFCI from the initial diagnosis to recovery.

## AUTHOR CONTRIBUTIONS

Blood samples were collected during experiments performed in laboratories at the University of Portsmouth and Catterick Garrison. Concentrations of biomarkers were analysed in laboratories at the University of Portsmouth and Loughborough University. Clare Eglin, Anthony Shepherd, Heather Massey, Sarah Hollis, Matthew Maley, Hugh Montgomery and Michael Tipton were involved in the concept and design of the work. Clare Eglin, Jennifer Wright, Anthony Shepherd, Heather Massey, Jonathan Towse, John Young, Matthew Maley, Stephen Bailey, Chris Wilkinson, Hugh Montgomery and Michael Tipton were involved the acquisition, analysis or interpretation of the data. Clare Eglin produced the first draft of the manuscript, and all authors revised it critically, approved the final version of the manuscript and agree to be accountable for all aspects of the work in ensuring that questions related to the accuracy or integrity of any part of the work are appropriately investigated and resolved. All persons designated as authors qualify for authorship, and all those who qualify for authorship are listed.

## CONFLICT OF INTEREST

None declared.

## Supporting information

Statistical Summary Document

## Data Availability

The data that support the findings of this study are available at: pure.port@ac.uk; https://doi.org/10.17029/de956bba-5b05-49b5-bfb1-f54945f9f367.

## 1

**TABLE A1:** Details of the enzyme‐linked immunosorbent assay kits used, the sensitivity, standard range, dilution factor and coefficient of variation (CV) for the various biomarkers.

					**% CV (SD)**		
**Biomarker**	**ELISA kit**	**Sensitivity**	**Standard range**	**Dilution**	**Baseline**	**Post‐heating**	**Post‐cooling**
TTPA (ng/ml)	Human plasminogen activator, tissue (tPA) ELISA kit, MyBioSource, USA	0.143	0.156–10	1	10.70 (10.71)	7.16 (16.81)	33.17 (29.23)
4‐HNE (ng/ml)	Human 4‐hydroxy‐2‐nonenal ELISA kit, MyBioSource, USA	0.1	31.25–2000	1	−3.72 (4.07)	−5.57 (7.59)	−8.30 (9.43)
Endothelin‐1 (pg/ml)	Human endothelin pan ELISA kit, Biorbyt, UK	<0.5	3.91–250	1	42.62 (44.9)	46.55 (113.75)	−36.29 (312.83)
IL‐6 (pg/ml)	Human interleukin 6 ELISA kit, LSBio, USA	<0.8	1.56–50	1	19.51 (39.8)	17.34 (15.14)	26.99 (43.50)
IL‐10 (pg/ml)	Human IL‐10 high sensitivity ELISA kit, Tecan, Switzerland	0.05	0.39–25	2	4.72 (67.09)	–	–
vWF (ng/ml)	Human von Willebrand factor ELISA kit, Abcam, UK	0.079	0.469–30	1000	4.04 (3.46)	–	–
Syndecan‐1 (ng/ml)	Human syndecan 1 ELISA Kit, Abcam, UK	4.94	8–256	2	15.33 (37.94)	–	–
SOD (ng/ml)	Human superoxide dismutase 1 ELISA Kit, Abcam, UK	0.04	0.08–5	40	7.81 (5.28)	–	–
Protein carbonyl (ng/ml)	Human protein carbonyl ELISA kit, Li StarFish, Italy	1.07	2–600	1	−3.47 (12.11)	–	–
Protein carbonyl (nmol/mg)	Protein carbonyl ELISA kit (Abcam, UK)	na	0.375–7.5	1	0	–	–
TNFα (pg/ml)	TNF‐alpha (TNF‐a) high sensitivity ELISA, Tecan, Switzerland	0.13	0.156–10	1	7.28 (5.70)	–	–
E‐selectin (ng/ml)	Human SE‐selectin/CD62E ELISA kit PicoKine, Boster Biological Technology, USA	0.3	1.6–50	5	19.91 (19.00)	–	–
Nitrotyrosine (ng/ml)	Human 3‐nitrotyrosine (3‐NT) ELISA kit, Biomatik, USA	2.56	0.156–10	1	−15.18 (235.8)	–	–

Abbreviations: CV, coefficient of variation; TTPA, tissue type plasminogen activator; 4‐HNE, 4‐hydroxy‐2‐noneal; IL‐6, interleukin‐6; IL‐10, interleukin‐10; vWF, von Willebrand Factor; SOD, superoxide dismutase; TNFα, tumour necrosis factor alpha.

**TABLE A2:** The percentage of samples with a coefficient of variation (CV) >15%, 10% below or above the standard curve, or with less than zero expression in baseline samples from the NFCI, COLD and CON groups for each biomarker.

**Biomarker**	**Percentage of samples with a CV >15%**			**Percentage of samples 10% below the standard curve**			**Percentage of samples 10% above the standard curve**			**Percentage of samples with less than zero expression**		
	**NFCI**	**COLD**	**CON**	**NFCI**	**COLD**	**CON**	**NFCI**	**COLD**	**CON**	**NFCI**	**COLD**	**CON**
Protein carbonyl	0	0	0	0	0	0	0	0	0	100	100	100
4‐HNE	14.3	0	0	0	0	0	0	0	0	0	0	0
Endothelin‐1	0	0	0	0	0	0	0	0	0	0	0	0
IL‐6	0	0	16.6	85.7	71.4	50	0	0	0	14.3	14.3	33.3
IL‐10	85.7	71.4	0	0	14.3	100	0	0	0	0	14.3	100
vWF	0	0	0	0	0	0	0	0	0	0	0	0
Syndecan‐1	57	14.3	0	0	0	0	0	14.3	16.7	0	0	16.7
SOD	14.3	0	0	0	0	0	0	0	0	0	0	33.3
TTPA	28.6	0	0	57.2	57.2	33.3	0	0	0	0	14.3	16.7
TNFα	0	28.6	0	0	0	0	0	0	0	0	0	0
E‐selectin	71.5	71.5	33.3	0	0	16.6	0	0	0	0	0	0
Nitrotyrosine	0	14.3	50	0	0	0	0	0	0	100	85.7	50

Abbreviations: CV, coefficient of variation; COLD, cold‐exposed control group; CON, non cold‐exposed control group; NFCI, individuals with chronic NFCI; 4‐HNE, 4‐hydroxy‐2‐noneal; IL‐6, interleukin‐6; IL‐10, interleukin‐10; vWF, von Willebrand Factor; SOD, superoxide dismutase; TTPA, tissue type plasminogen activator; TNFα, tumour necrosis factor alpha.

**TABLE A3:** The percentage of samples with a coefficient of variation (CV) >15%, 10% below or above the standard curve, or with less than zero expression in post‐heating and cooling plasma samples for 4‐HNE, endothelin‐1, IL‐6 and TTPA in the NFCI, COLD and CON participant groups.

**Biomarker**	**Percentage of samples with a CV >15%**			**Percentage of samples 10% below the standard curve**			**Percentage of samples 10% above the standard curve**			**Percentage of samples with less than zero expression**		
	**NFCI**	**COLD**	**CON**	**NFCI**	**COLD**	**CON**	**NFCI**	**COLD**	**CON**	**NFCI**	**COLD**	**CON**
Post‐heating												
4‐HNE	9.1	20	10	0	0	0	0	0	30	0	0	0
Endothelin‐1	0	40	30	0	10	10	0	0	0	0	30	40
IL‐ 6	36.4	30	30	18.2	50	0	0	0	0	0	10	10
TTPA	27.3	20	10	0	10	10	0	0	0	0	0	0
4‐HNE	11.1	0	14.3	0	0	0	0	0	0	0	0	0
Post‐cooling												
Endothelin‐1	55.6	12.5	28.6	22.2	12.5	14.3	0	0	14.3	22.2	75	42.9
IL‐ 6	66.7	75	28.6	0	0	0	0	0	0	0	0	0
TTPA	88.9	37.5	42.9	0	0	0	0	0	0	0	0	0

Abbreviations: CV, coefficient of variation; COLD, cold‐exposed control group; CON, non cold‐exposed control group; NFCI, individuals with chronic NFCI; 4‐HNE, 4‐hydroxy‐2‐noneal; IL‐6, interleukin‐6; vWF, von Willebrand Factor; TTPA, tissue type plasminogen activator.
